# New report of two patients with mosaic trisomy 9 presenting unusual features and longer survival

**DOI:** 10.1590/S1516-31802011000600010

**Published:** 2011-12-01

**Authors:** Paulo Ricardo Gazzola Zen, Rafael Fabiano Machado Rosa, Rosana Cardoso Manique Rosa, Carla Graziadio, Giorgio Adriano Paskulin

**Affiliations:** I PhD. Adjunct Professor of Clinical Genetics, Professor of the Postgraduate Pathology Program and Clinical Geneticist, Universidade Federal de Ciências da Saúde de Porto Alegre (UFCSPA), and Complexo Hospitalar Santa Casa de Porto Alegre (CHSCPA), Porto Alegre, Rio Grande do Sul, Brazil.; II MD. Postgraduate Student and Clinical Geneticist, Universidade Federal de Ciências da Saúde de Porto Alegre (UFCSPA), and Complexo Hospitalar Santa Casa de Porto Alegre (CHSCPA), Porto Alegre, Rio Grande do Sul, Brazil.; III MD. Pediatrician and Postgraduate Student, Universidade Federal de Ciências da Saúde de Porto Alegre (UFCSPA), Porto Alegre, Rio Grande do Sul, Brazil.; IV MD. Assistant Professor of Clinical Genetics and Clinical Geneticist, Universidade Federal de Ciências da Saúde de Porto Alegre (UFCSPA), and Complexo Hospitalar Santa Casa de Porto Alegre (CHSCPA), Porto Alegre, Rio Grande do Sul, Brazil.; V PhD. Associated Professor of Clinical Genetics, Professor of the Postgraduate Pathology Program, Clinical Geneticist and Cytogeneticist, Universidade Federal de Ciências da Saúde de Porto Alegre (UFCSPA), and Complexo Hospitalar Santa Casa de Porto Alegre (CHSCPA), Porto Alegre, Rio Grande do Sul, Brazil.

**Keywords:** Mosaicism, Chromosomes, human, pair 9, Chromosome aberrations, Goldenhar syndrome, Survivorship (Public health), Mosaicismo, Cromossomos humanos par 9, Aberrações cromossômicas, Síndrome de Goldenhar, Sobrevida

## Abstract

**CONTEXT:**

Mosaic trisomy 9 is considered to be a rare chromosomal abnormality with limited survival. Our objective was to report on two patients with mosaic trisomy 9 presenting unusual findings and prolonged survival.

**CASE REPORTS:**

The first patient was a boy aged six years and five months presenting weight of 14.5 kg (< P3), height of 112 cm (P10), head circumference of 49 cm (P2), prominent forehead, triangular and asymmetric face, thin lips, right microtia with overfolded helix, small hands, micropenis (< P10), small testes and hallux valgus. His lymphocyte karyotype was mos 47,XY,+9[4]/46,XY[50]. Additional cytogenetic assessment of the skin showed normal results. The second patient was a two-year-old girl who was initially assessed at five months of age, when she presented weight of 5.3 kg (< P3), height of 61.5 cm (P2-P10), head circumference of 40.5 cm (P25), sparse hair, micrognathia, right ear with overfolded helix and preauricular pit, triphalangeal thumbs and sacral dimple. She also had a history of congenital heart disease, hearing loss, hypotonia, delayed neuropsychomotor development and swallowing disorder. Her lymphocyte karyotype was mos 47,XX,+9[3]/46,XX[69]. Both patients had unusual clinical findings (the first, hemifacial hypoplasia associated with microtia, with a phenotype of oculo-auriculo-vertebral spectrum, and the second, triphalangeal thumbs and hearing loss) and survival greater than what is usually described in the literature (< 1 year). Further reports will be critical for delineating the clinical features and determining the evolution of patients with mosaic trisomy 9.

## RESUMO

**CONTEXTO:**

A trissomia do cromossomo 9 em mosaico é considerada uma anormalidade cromossômica rara e com limitada sobrevida. Nosso objetivo foi realizar o relato de dois pacientes com trissomia do 9 em mosaico, apresentando achados não usuais e sobrevida prolongada.

**RELATO DE CASOS:**

O primeiro paciente era um menino de seis anos e cinco meses apresentando peso de 14,5 kg (< P3), altura de 112 cm (P10), perímetro cefálico de 49 cm (P2), proeminência frontal, face triangular e assimétrica, lábios finos, microtia à direita com hélix sobredobrado, mãos pequenas, micropênis (< P10), testículos pequenos e hálux valgo. Seu cariótipo em linfócitos foi mos 47,XY,+9[4]/46,XY[50]. O estudo citogenético complementar da pele foi normal. A segunda paciente era uma menina de dois anos, avaliada inicialmente aos cinco meses, quando apresentava peso de 5,3 kg (< P3), estatura de 61,5 cm (P2-P10), perímetro cefálico de 40,5 cm (P25), cabelos esparsos, micrognatia, orelha direita com sobredobramento do hélix e fosseta pré-auricular, polegares trifalangeanos e fosseta sacral. Ela possuía também história de cardiopatia congênita, perda auditiva, hipotonia, atraso do desenvolvimento neuropsicomotor e distúrbio da deglutição. Seu cariótipo de linfócitos foi mos 47,XX,+9[3]/46,XX[69]. Os dois pacientes apresentam achados clínicos não usuais (o primeiro, hipoplasia hemifacial associada à microtia lembrando um fenótipo de espectro óculo-aurículo-vertebral, e o segundo, polegares trifalangeanos e perda auditiva) e uma sobrevida maior àquela usualmente descrita na literatura (< 1 ano). Mais relatos serão fundamentais para delinear o quadro clínico e determinar a evolução de pacientes com trissomia do 9 em mosaico.

## INTRODUCTION

Trisomy 9 is considered to be a rare chromosomal abnormality. Since the first descriptions, which were made in 1973, more than 50 patients have been described in the literature, although reports from Brazil are uncommon.^1^ Trisomy 9 has been reported both alone and, especially, in mosaic with a normal cell line.^2,3^ However, the patients usually present similar clinical features, independent of the presence of mosaicism, characterized by growth retardation, mental deficiency and brain, facial, cardiac, renal and skeletal abnormalities.^2^

It is interesting to note that normal chromosomal cells are identified in patients with a previous diagnosis of trisomy 9 alone when a great number of cells are analyzed through cytogenetic molecular techniques such as fluorescence *in situ* hybridization, as demonstrated by Cantú et al.^4^ Nevertheless, the survival of patients with mosaicism is longer than that of individuals with trisomy 9 alone. While trisomy 9 patients survive for a mean of 20 days,^3^ the follow-up on patients with mosaicism may reach beyond the first year of life.^3,5,6^

Thus, we report here on two patients with mosaic trisomy 9 presenting uncommon clinical features and a longer follow-up. These patients were evaluated by medical geneticists at the Clinical Genetics Sector of the Universidade Federal de Ciências da Saúde de Porto Alegre (UFCSPA) and Complexo Hospitalar Santa Casa de Porto Alegre (CHSCPA), a service that has existed in the State of Rio Grande do Sul, Brazil, for nearly 35 years.

## CASE REPORTS

### Patient 1

The patient was a Caucasian boy of six years and five months of age, the third son of a non-consanguineous couple aged 38 years (mother) and 47 years (father). The family history was unremarkable. The child was born by vaginal delivery, with 30 weeks of gestation, weighing 1,660 g (P50-90) and with an Apgar score of 9 at the fifth minute. The mother reported that she had used diazepam (10 mg three times a week), smoked 40 cigarettes per day and consumed alcohol every day throughout her pregnancy. After the birth, the child evolved with inadequate weight gain and jaundice, with the need for phototherapy.

The boy underwent left orchidopexy at the age of one year and eight months. His neuropsychomotor development was delayed, since he started to walk without support at the age of two year and six months, spoke his first words at the age of five years and, at this age, still did not have sphincter control. Additional evaluation through a brain computed tomography scan showed normal results.

**Figure 1:**
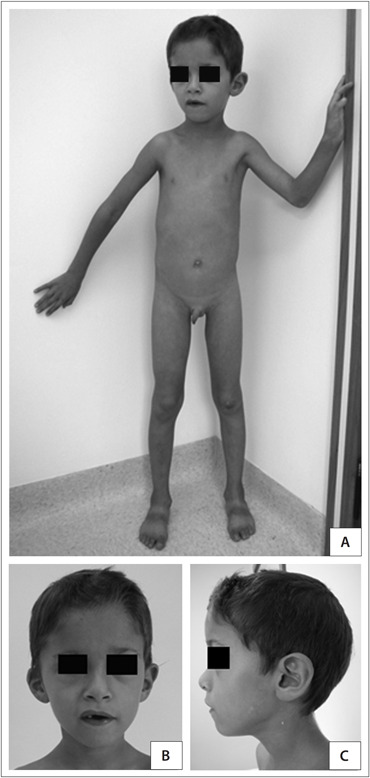
Patient 1: six-year-old boy with mosaic trisomy 9.

At a physical examination performed at the age of six years and five months, the patient presented a weight of 14,500 g (< P3), height of 112 cm (P10), head circumference of 49 cm (P2), hand length of 11.5 cm (< P3), middle finger length of 4.5 cm (< P3), prominent forehead, triangular and asymmetrical face (right hemiface was smaller), smooth philtrum, thin lips, deviation of the labial commissure during speech, microtia of the right ear with overfolded helix, diastasis recti, micropenis [penis length of 4 cm (< P10)], small testicles and hallux valgus (Figure 1). He presented a heart murmur, but echocardiography showed normal results.

**Figure 2:**
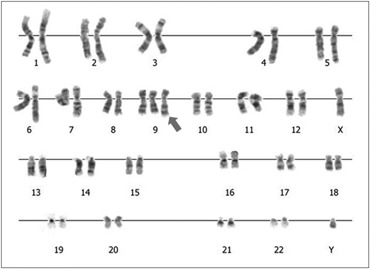
Cytogenetic analysis of the patient #1.

Cytogenetic analysis on GTG-banding karyotypes showed mosaicism between a normal male chromosomal lineage and another with trisomy 9: mos 47,XY,+9[4]/46,XY[50] (Figure 2). The karyotype analysis on fibroblasts was normal: 46,XY[59].

### Patient 2

The patient was a two-year-old Caucasian girl, the daughter of parents aged 37 years (mother) and 54 years (father). The gestational and family data were unavailable. An atrial septal defect associated with a ventricular septal defect and patent ductus arteriosus were diagnosed at birth. She also presented a swallowing disorder and gastroesophageal reflux.

At a physical examination, at the age of five months, she presented a height of 61.5 cm (P2-10), weight of 5.3 kg (< P3), head circumference of 40.5 cm (P25), sparse hair, micrognathia, right ear with an overfolded helix and a preauricular pit, long thumbs, sacral dimple and long and tapered toes. Radiographic evaluation of the hands and feet revealed triphalangeal thumbs. The child also had a history of hearing loss (with abnormal auditory evoked potential), significant hypotonia and neuropsychomotor delay. Her electroencephalogram was normal, and she did not present seizures. She was using an auditory prosthesis and was being followed up with physiotherapy and speech therapy.

Cytogenetic evaluation from peripheral blood on GTG-banding karyotypes showed mosaic trisomy 9: mos 47,XX,+9[3]/46,XX[69]. Fibroblast evaluation was not performed.

## DISCUSSION

Mosaic trisomy 9 is an infrequent condition associated with limited survival. Our review of the PubMed, Scirus, Embase, Cochrane Library, Lilacs and SciELO databases using specific descriptors can be seen in Table 1. An additional chromosome 9 in blood tests, which was not detected in other tissues such as the skin,^6^ as observed with patient 1, or only identified in fibroblasts,^7^ has been described in the literature. Schwartz et al., evaluating different tissues, such as hepatic, pulmonary and heart cells, demonstrated high variability of the mosaicism found in a single individual with mosaic trisomy 9.^8^

**Table 1:** Results from our review using descriptors for the main features observed in our patients

Database*	Search strategy	Results
PubMed	"Chromosomes, Human, Pair 9" AND "Mosaicism" AND "Trisomy"	42 case reports 11 reviews
	"Chromosomes, Human, Pair 9" AND "Mosaicism" AND "Trisomy" AND "facial asymmetry"	1 case report
	"Chromosomes, Human, Pair 9" AND "Mosaicism" AND "Trisomy" AND "thumb"	1 case report
	"Chromosomes, Human, Pair 9" AND "Mosaicism" AND "Trisomy" AND "survival"	1 case report 1 review
Scirus	"Chromosomes, Human, Pair 9" AND "Mosaicism" AND "Trisomy"	61 articles
	"Chromosomes, Human, Pair 9" AND "Mosaicism" AND "Trisomy" AND "facial asymmetry"	1 case report
	"Chromosomes, Human, Pair 9" AND "Mosaicism" AND "Trisomy" AND "thumb"	1 case report
	"Chromosomes, Human, Pair 9" AND "Mosaicism" AND "Trisomy" AND "survival"	4 articles
Embase	"Chromosomes, Human, Pair 9" AND "Mosaicism" AND "Trisomy"	20 articles 3 reviews 1 letter 1 conference abstract 1 conference paper
	"Chromosomes, Human, Pair 9" AND "Mosaicism" AND "Trisomy" AND "facial asymmetry"	0 articles
	"Chromosomes, Human, Pair 9" AND "Mosaicism" AND "Trisomy" AND "thumb"	0 articles
	"Chromosomes, Human, Pair 9" AND "Mosaicism" AND "Trisomy" AND "survival"	0 articles

* No results were observed using the same search strategies in the Cochrane Library, Lilacs and SciELO databases.

The clinical variability observed in mosaic trisomy 9 may result either from a varying degree of mosaicism or from the different tissues involved. Moreover, it may be associated with the existence of undetected uniparental disomy in the normal chromosomal lineage, which will have occurred during chromosomal rescue.^6^ This has only been investigated in a few cases of mosaic trisomy 9, and in our review, we found only one report with this abnormality.^6,7^ Additionally, uniparental disomy of chromosome 9 alone has been rarely reported. All the cases were of maternal uniparental disomy, and the patients described presented features of autosomal recessive diseases, such as cartilage-hair hypoplasia and Leigh syndrome, for which the genes are located in chromosome 9.^9-11^

Facial abnormalities are common in cases of mosaic trisomy 9. However, most of the abnormalities observed in our patients differed from those most frequently described in the literature, which include high or narrow forehead, microcephaly, short and upslanting palpebral fissures, deep-set eyes, microphthalmus, epicanthal folds, hypertelorism, broad nasal bridge, bulbous nose, high arched palate, cleft lip or palate, micrognathia and large fontanelles.^2^ Ear abnormalities, as identified in our patients (with the exception of the preauricular pit observed in patient 2), are considered to be very common and have been described in more than 90% of such patients.^3^ On the other hand, facial asymmetry caused by hemifacial hypoplasia is an uncommon feature,^2,4,6^ and this may be related to the mosaicism presented by the patients.^6^ The association of this feature with ipsilateral microtia observed in patient 1 may also suggest the presence of the phenotype of another condition, the oculo-auriculo-vertebral spectrum (OAVS), also known as hemifacial microsomia or Goldenhar syndrome. This is considered to be a phenotype characterized by variable clinical and etiological features. Although most cases have been sporadic and have not presented any known cause, different chromosomal abnormalities have been described in subjects with this phenotype, and mosaic trisomy 9 is one of them^12^ (Table 1). Interestingly, the patient described by Willatt et al., with uniparental disomy of chromosome 9 in the normal chromosomal lineage, presented facial asymmetry.^6^ However, no case of uniparental disomy of chromosome 9 alone has been reported with these features of OAVS.^9-11^

Cardiac abnormalities are frequent (around 70% of the cases), and the most common types correspond to those presented by patient 2, i.e. ventricular and atrial septal defects and patent ductus arteriosus. Genitourinary abnormalities affect 73% of the patients, and micropenis and cryptorchidism, as observed in patient 1, are very common among male subjects.^3^ In relation to skeletal malformations, dislocations and bone absences involving especially the hips, knees, pelvis, ribs, hands and feet are frequent in cases of mosaic trisomy 9.^3^ Nevertheless, neither of our patients presented such abnormalities. Our attention was drawn to the finding of triphalangeal thumbs observed in patient 2, because this was an abnormality that had not previously been described in cases of mosaic trisomy 9 (Table 1) or even in uniparental disomy of chromosome 9. In these cases, the impairment of the thumbs consisted only of limited abduction. Gastrointestinal abnormalities are also infrequent, and the swallowing disorder and gastroesophageal reflux observed in patient 2 have only been described in a few cases.^3^

Neuropsychomotor delay and mental retardation are common features among survivors. However, there are also descriptions in the literature of individuals with mosaicism and normal development.^3^ In our cases, both patients presented neuropsychomotor delay. However, we cannot rule out the possibility that the gestational exposure to tobacco and alcohol and the prematurity seen in the case of the first patient may have influenced his development. Central nervous system abnormalities were not observed in our patients, but abnormalities such as Dandy-Walker malformation have been identified in trisomy 9 subjects without mosaicism. We did not find any descriptions of hearing loss in the literature, among patients with mosaic trisomy 9, as observed in patient 2.

Neither of our patients died. However, the mean survival of subjects with mosaic trisomy 9 is still not known, because, as highlighted by other authors,^5^ a significant proportion of the patients reported with this condition were not dead and were very young at the time of their descriptions (Table 1). Nevertheless, survival beyond the first year is considered uncommon.^3,5,6^ It seems that patient 1 has good survival prospects, because he does not present major abnormalities such as congenital heart defect or respiratory and gastrointestinal complications. We cannot rule out the possibility that this longer survival may be related to the degree of mosaicism observed or even to the tissue distribution of the trisomy. It is possible that in such cases the trisomic cells may be especially present in "less prime" tissues. In patient 1, for example, the mosaicism was detected only in lymphocytes and not in fibroblasts. Another point to be considered is in relation to the improvement in healthcare for such patients that has come about over the past decades, which may present an influence on their survival.

## CONCLUSION

Mosaic trisomy 9 is a rare chromosomal abnormality that appears to present significant phenotypic variability. Patients showing improved survival have been described in the literature. Thus, more reports, especially with long-term follow-up, will be fundamental both to better delineate the clinical picture and to determine the evolution and follow-up of mosaic trisomy 9. Such information will be of great importance for parents and relatives of subjects with this condition.

## References

[1] Moskovitz M, Brener D, Annick RR (2006). Dental management of a child with trisomy 9 mosaicism: a case report. Pediatr Dent.

[2] Arnold GL, Kirby RS, Stern TP, Sawyer JR (1995). Trisomy 9: review and report of two new cases. Am J Med Genet.

[3] Wooldridge J, Zunich J (1995). Trisomy 9 syndrome: report of a case with Crohn disease and review of the literature. Am J Med Genet.

[4] Cantú ES, Eicher DJ, Pai GS, Donahue CJ, Harley RA (1996). Mosaic vs. nonmosaic trisomy 9: report of a liveborn infant evaluated by fluorescence in situ hybridization and review of the literature. Am J Med Genet.

[5] Okumura A, Hayakawa F, Kato T, Kuno K, Watanabe K (2000). Two patients with trisomy 9 mosaicism. Pediatr Int.

[6] Willatt LR, Davison BC, Goudie D (1992). A male with trisomy 9 mosaicism and maternal uniparental disomy for chromosome 9 in the euploid cell line. J Med Genet.

[7] Lindor NM, Michels VV, Jalal S, Shaughnessy W (1995). Trisomy 9 mosaicism in a child with tethered cord. Clin Dysmorphol.

[8] Schwartz S, Ashai S, Mejboom EJ (1989). Prenatal detection of trisomy 9 mosaicism. Prenat Diagn.

[9] Sulisalo T, Mäkitie O, Sistonen P (1997). Uniparental disomy in cartilage-hair hypoplasia. Eur J Hum Genet.

[10] Tiranti V, Lamantea E, Uziel G (1999). Leigh syndrome transmitted by uniparental disomy of chromosome 9. J Med Genet.

[11] Slater HR, Ralph A, Daniel A, Worthington S, Roberts C (2000). A case of maternal uniparental disomy of chromosome 9 diagnosed prenatally and the related problem of residual trisomy. Prenat Diagn.

[12] Wilson GN, Barr M (1983). Trisomy 9 mosaicism: another etiology for the manifestations of Goldenhar syndrome. J Craniofac Genet Dev Biol.

